# Caregiver Burden and Support for People with Neurological Disorders: Findings from a Polish Cross-Sectional Study

**DOI:** 10.3390/jcm15020674

**Published:** 2026-01-14

**Authors:** Małgorzata Pasek, Zofia Strzesak, Anna Goździalska, Małgorzata Jochymek

**Affiliations:** 1 Department of Nursing, Faculty of Health Sciences, University of Applied Sciences in Tarnow, 33-100 Tarnow, Poland; m_pasek@atar.edu.pl (M.P.); zosia19999@op.pl (Z.S.); 2 Faculty of Health Sciences, Andrzej Frycz Modrzewski Cracow University, 30-705 Krakow, Poland; mjochymek@uafm.edu.pl

**Keywords:** neurological diseases, informal caregivers, social support, Berlin Social Support Scales (BSSS), respite care

## Abstract

**Background/Objectives**: Neurological diseases are a major cause of long-term disability and dependence. In Poland, as in many countries, informal caregivers provide most long-term care for individuals with chronic and progressive neurological conditions. Although essential, this role is associated with substantial physical, psychological, and social burden. This study aimed to assess the scope and nature of support provided by caregivers to people with neurological diseases and to identify factors associated with differences in support and caregiver burden. **Methods**: A cross-sectional quantitative study was conducted using a CAWI survey. The sample included 104 informal caregivers of adults with various neurological conditions. An author-designed questionnaire and the “Actually Provided Support” subscale of the Berlin Social Support Scales (BSSS) were used. Nonparametric statistical tests were applied (*p* < 0.05). **Results**: Caregivers provided a high level of support, particularly emotional and instrumental support, while informational support was less intensive. Women more frequently reported high emotional and instrumental support. Higher buffering–protective support was more common among caregivers aged over 45 years. The most frequently reported difficulties were psychological fatigue (70.9%) and physical fatigue (60.2%), indicating a substantial caregiving burden. Key barriers included limited access to reimbursed healthcare services and the lack of temporary replacement in caregiving. Caregivers most often indicated the need for respite care and better access to information and education. **Conclusions**: Informal caregivers play a crucial role in the daily functioning of people with neurological diseases, despite high burden and insufficient systemic support. Expanding respite care, improving access to information, and better coordination of healthcare services are urgently needed.

## 1. Introduction

Neurological conditions have long ranked among the leading causes of disability, long-term dependence on others, and loss of independence. According to the World Health Organization, such conditions account for around 10% of the global burden of disease, and their prevalence is increasing as populations age. Current projections suggest that, over the coming decades, the number of people requiring daily care due to neurological disorders may rise by as much as 40–60%. This trend places heavy demands on healthcare systems, social care services, and the families who most commonly assume the role of primary carers [1,2,3].

In Poland, as in many other European countries, family carers provide the vast majority of long-term support for people with severe neurological deficits. European estimates indicate that as much as 80–85% of all long-term care is delivered informally: by relatives, friends and neighbours. Although this form of care is largely “invisible” to the system, its economic significance is considerable; in some EU member states, its estimated value reaches 2–3% of GDP [4,5,6].

Caring for patients with neurological conditions involves several distinctive characteristics that intensify the load placed on carers [7]. First, most neurological disorders are chronic and progressive. Second, many patients experience a combination of motor impairments, cognitive decline, behavioural changes and communication difficulties. Third, the clinical course is often unpredictable, with periods of stability interspersed with sudden deterioration. Finally, many neurological diseases entail a gradual loss of the patient’s former identity, which carers often find particularly painful and emotionally taxing [8,9,10,11].

Research conducted among populations living with dementia, Parkinson’s disease, multiple sclerosis, epilepsy, and stroke shows that carers experience exceptionally high levels of strain. Brodaty and Donkin describe this burden as “a constant battle on two fronts”: on the one hand, against the symptoms of the illness, and on the other, against the carer’s own fatigue, uncertainty, anxiety, and sense of losing control [12]. Among carers supporting people after a stroke, physical challenges tend to dominate, particularly those related to transfers, personal hygiene, and feeding. In dementia, by contrast, the main burden arises from cognitive decline and behavioural disturbances. In Parkinson’s disease, the nature of strain shifts with the progression of symptoms from predominantly motor problems to escalating neuropsychiatric complications [9,10].

Theoretical models have played an important role in understanding caregiver burden. Pearlin’s Stress Process Model, for instance, posits that strain results from the interaction between primary stressors (such as symptoms of the illness, loss of independence, and behavioural problems), secondary stressors (including role conflict and financial strain), and available resources, such as social support and coping strategies. Empirical findings demonstrate that a combination of strong primary stressors and insufficient social support leads to chronic emotional overload [12]. Another influential framework, Lazarus and Folkman’s transactional model of stress and coping, holds that the caregiver’s subjective appraisal of the situation determines both their coping strategies and the intensity of stress [4,13].

Despite a substantial body of research, studies involving mixed neurological populations remain scarce, even though such groups more closely mirror clinical reality, as patients commonly present with overlapping motor, cognitive and behavioural impairments. This gap is particularly visible in Central and Eastern Europe, where system-level support for family carers is less developed than in Scandinavian or Western European contexts. In Poland, for example, respite care is difficult to access, psychological support for carers is limited, and support pathways are opaque and poorly coordinated [6,14,15,16,17].

Against this backdrop, it has become increasingly important to investigate both the scope and the nature of support provided by carers of people with neurological conditions, as well as the factors that modulate caregiver burden. Our study concentrates on the active role of carers in the care process, analysing the forms of support that they provide on a daily basis and the conditions that shape these caregiving practices.

## 2. Materials and Methods

### 2.1. Study Design and Research Methods

This study was designed as a cross-sectional quantitative investigation aimed at assessing caregiver burden and the scope of support provided by caregivers to individuals with neurological conditions. Data were collected using a diagnostic survey method, which enabled the examination of both subjective perceptions and objective aspects of the caregiving situation. The CAWI method (Computer-Assisted Web Interviewing) was employed, using an online questionnaire completed independently by participants via a computer or mobile devices.

Eligibility criteria included: age ≥ 18 years; provision of unpaid care (informal caregiving) to a person with a neurological condition for at least one month; and the ability to complete the questionnaire independently. Eligibility was verified through questionnaire items. The sole exclusion criterion was lack of informed consent.

The research instrument was a 44-item questionnaire comprising both an author-deigned section and a standardised scale addressing the main research objective. The questionnaire was administered electronically in a manner ensuring respondent anonymity.

The author-developed section included sociodemographic characteristics, caregiving workload, and proxy reporting, whereby caregivers provided information on the care recipient’s health status, functional capacity, and the relationship between these variables. The standardised instrument used was the Berlin Social Support Scales (BSSS), specifically the caregiver version, Actually Provided Support. This subscale assesses the scope and type of support provided by caregivers, including emotional, instrumental, and informational support in daily care. Responses were rated on a four-point Likert scale, with higher scores indicating greater levels of support. The internal consistency of the scale was high (Cronbach’s α = 0.86). In addition, selected information regarding the care recipients’ functional status and care needs was collected using proxy reporting by caregivers. This approach was adopted due to the high prevalence of cognitive, communication, and functional impairments among people with neurological conditions, which often preclude reliable self-reporting.

Statistical analyses were performed using STATISTICA 10 (StatSoft) Cracov. Chi-square analyses indicated gender-related differences in selected types of social support. Descriptive statistics were calculated, and exploratory non-parametric analyses were conducted to examine associations between caregiving characteristics and selected sociodemographic variables. For chi-square analyses, effect sizes were calculated using Cramer’s V to assess the strength of associations. In cases where contingency tables contained zero or very small cell counts, categories were merged where conceptually justified, and Fisher’s exact test was applied to ensure robust statistical inference. Statistical significance was set at *p* < 0.05.

### 2.2. Study Organisation and Procedure

This study was non-interventional and involved the anonymous completion of an online questionnaire. No personally identifiable data were collected, and participation did not entail any risk or burden for respondents. In accordance with Polish law and the regulations governing research involving human participants, studies of this type do not require approval from a bioethics committee.

The questionnaire was administered via the dedicated online platform Google Forms, which enabled the automatic recording and archiving of data. Before deciding whether to participate, potential respondents were provided with information regarding the purpose of this study, as well as assurances of anonymity and voluntary participation. Upon providing informed consent, participants were granted access to the first question of the questionnaire. If consent was not given, the form automatically closed without allowing access to the survey items.

Participants were free to withdraw from this study at any stage without providing a reason. Data were analysed exclusively in aggregated form.

### 2.3. Participants

This study included 104 informal caregivers of individuals with various neurological conditions. The sample was heterogeneous with respect to age, educational level, place of residence, and economic status, which allowed for the capture of a broad spectrum of caregiving experiences and the identification of factors associated with increased or reduced caregiver burden. The sociodemographic characteristics of the participants are presented in Table 1.

The caregiver sample was predominantly female (86.4%), with more than half of participants aged 45 years or younger (55.8%). Over half of the respondents reported having higher (tertiary) education (54.4%). A considerable proportion resided in rural areas, where access to caregiving and rehabilitation services is often limited, which may have contributed to increased caregiver burden.

## 3. Results

The caregivers participating in this study provided daily, long-term care to individuals with chronic and progressive conditions that impaired patients’ functioning and increased the extent of assistance required. The care recipients represented a heterogeneous group with respect to the type of condition, stage of disease progression, and associated health consequences. The majority of patients had severe neurological or neurodegenerative disorders, which frequently led to reduced independence, functional decline, and an increased risk of complications. The socio-clinical characteristics of the care recipients are presented in Table 2.

Caregiving for a close relative required carers to manage nearly all aspects of the care recipient’s daily life, including assistance with personal hygiene, medication administration, and mobility, as well as, in many cases, constant supervision. The nature and progression of the care recipients’ conditions determined both the scope of caregiving responsibilities and the level of caregiver fatigue, providing essential context for the interpretation of the subsequent findings.

**Figure 1:**
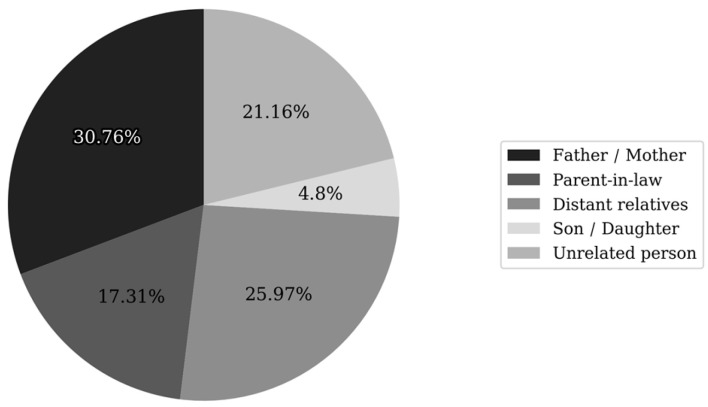
Relationship between the caregiver and the care recipient.

The caregivers most commonly provided care for a parent (30.76%, n = 32) or for more distant relatives (25.97%, n = 27). For 21.16% of respondents (n = 22), the care recipient was not a family member, while 17.31% (n = 18) were caring for a parent-in-law (Figure 1)

Nearly 44% of caregivers did not reside with their care recipients, while the remainder lived with them either permanently (38.8%) or intermittently (17.5%). The vast majority of respondents (82.5%) reported receiving assistance from others in the provision of care. The most frequently identified sources of support were spouses (42.4%), siblings (37.7%), children (14.1%), institutional services or staff (15.3%), as well as acquaintances (11.8%) and friends (11.8%).

Despite this, 40.8% of participants (n = 42) reported not using any form of institutional support. Among those who did, one or more types of assistance were utilised, most commonly financial support (50.5%), a care allowance (43.7%), a caregiver benefit (20.4%), and formal care services (10.7%).

**Figure 2:**
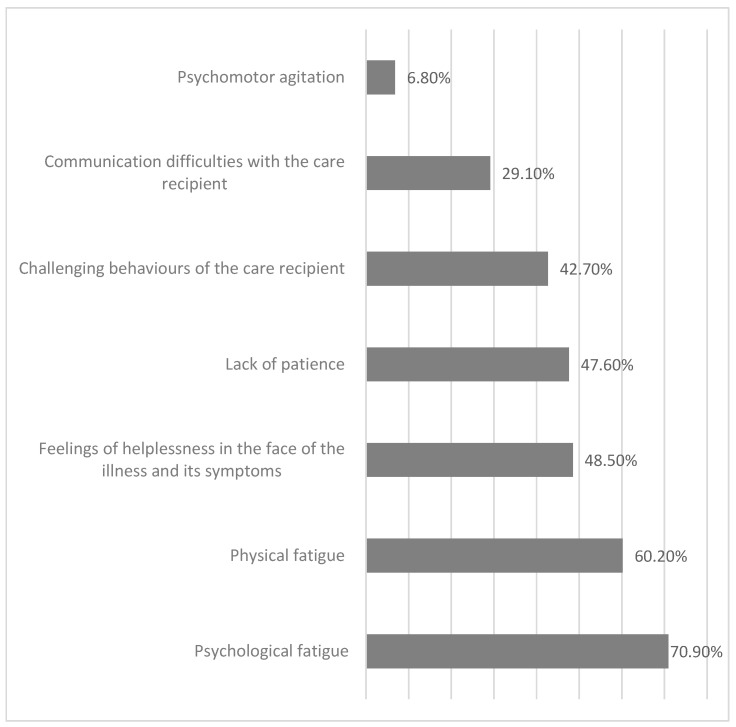
Difficulties reported by caregivers in daily provision of care.

Caregivers also reported difficulties encountered in the course of daily care, often selecting more than one response (Figure 2). The most prevalent burden was psychological fatigue (70.9%), with many respondents reporting a persistent sense of tension and responsibility. Physical fatigue was the second most frequently reported burden (60.2%), attributable to the physical demands of caregiving tasks and repetitive daily activities.

The analysis showed that among younger caregivers (aged 45 years or younger), a statistically significant association was observed specifically with feelings of helplessness in response to illness and its symptoms. No significant relationships were found between the other reported caregiving difficulties and caregivers’ gender, age, receipt of support from others, or use of financial assistance.

This study also identified external factors that hinder caregiving for individuals with disabilities. The most frequently reported barriers were limited access to healthcare services reimbursed by the National Health Fund (NFZ) (47.6%, n = 49) and the absence of respite care or someone who could temporarily assume caregiving responsibilities (38.8%, n = 40). Fewer than one in three caregivers identified additional external barriers to care, such as architectural barriers (32.0%, n = 33), insufficient information about available forms of support (30.1%, n = 31), or other factors. These responses suggest that caregivers often function in an environment that fails to provide adequate systemic support, thereby intensifying the burden associated with daily caregiving.

**Figure 3:**
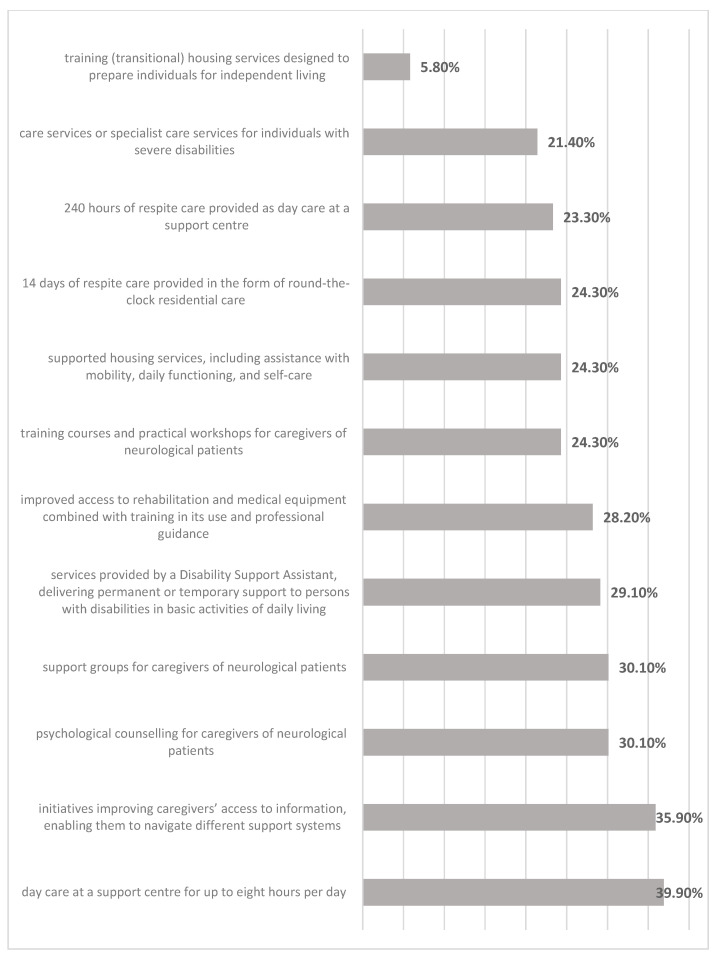
Preferred sources of support for relieving caregiving burden.

With regard to potential forms of relief from caregiving responsibilities, respondents most frequently expressed interest in day care services at a support centre for up to eight hours per day (36.9%, n = 38), as well as initiatives designed to improve caregivers’ access to information that would enable them to navigate different support systems, funding schemes, and benefits and thereby enhance the quality of care (35.9%, n = 37). Slightly lower levels of interest were reported for psychological counselling for caregivers of neurological patients (30.1%, n = 31), support groups for caregivers of neurological patients (30.1%, n = 31), and services provided by a Disability Support Assistant, which include permanent or temporary assistance with basic activities of daily living necessary for social, occupational, or educational participation (29.1%, n = 30). Improved access to rehabilitation and medical equipment, combined with training on how to use it and professional guidance, was also indicated by a similar proportion of respondents (28.2%, n = 29). Fewer than one in four caregivers expressed interest in other forms of relief from caregiving responsibilities (Figure 3).

Women were significantly more likely to express interest in support groups for caregivers of neurological patients (*p* = 0.0440). Caregivers who received financial support more frequently reported interest in a 14-day period of respite care provided as round-the-clock residential care (*p* = 0.0498). By contrast, caregivers who did not receive financial support more often preferred psychological counselling for caregivers of neurological patients (*p* = 0.0054). No significant associations were observed between the potential use of support resources and caregivers’ age, educational attainment, receipt of assistance from others, or use of financial benefits.

A key component of this study was the analysis of the standardised Berlin Social Support Scales (BSSS). The findings indicated that caregivers provided their care recipients with a relatively high degree of support. Mean scores were 21.44 for emotional support, 7.26 for instrumental support, and 4.19 for informational support (Table 3).

The emotional social support currently provided by caregivers was predominantly rated as high (68.0%, n = 70), with the remaining respondents reporting a moderate level (32.0%, n = 33). Similarly, nearly two thirds of caregivers reported providing a high level of instrumental support. Specifically, 67.0% of caregivers (n = 69) indicated a high level of instrumental support, 28.1% (n = 30) a moderate level, and only 3.9% (n = 4) a low level. Informational social support was reported as high by 44.7% of caregivers (n = 46) and as moderate by 37.9% (n = 39), while 17.5% (n = 18) indicated a low level of informational support. Satisfaction with the social support currently received was most frequently rated as moderate (43.7%, n = 45), followed by high (31.1%, n = 32), while one quarter of caregivers reported low satisfaction (25.2%, n = 26).

Female caregivers more frequently reported high levels of emotional social support compared to men (χ^2^ =7.738, *p* =0.0054; Table 4). However, sensitivity analyses using Fisher’s exact test suggested that this association should be interpreted with caution due to sparse data in some categories. Chi-square analyses suggested a gender-related difference in levels of instrumental social support (χ^2^ = 7.175, *p* = 0.0277; Table 4). This association did not retain statistical significance in sensitivity analyses using Fisher’s exact test.

The analysis of the relationship between gender and levels of support provision indicated that women more frequently reported high levels of emotional and instrumental support. This pattern is consistent with findings from studies conducted in other caregiver populations, in which women tend to be more involved in activities related to direct caregiving and the organisation of care. This may be explained both by socially entrenched gender role divisions and by differences in caregiving practices between women and men.

It is important to note, however, that the observed differences do not necessarily reflect variations in the quality of care provided, but rather the gendered distribution of caregiving tasks. Men may be more involved in other forms of support that are not adequately captured by the measurement tool used in this study. The findings therefore underscore the importance of applying a gender-sensitive perspective when designing educational and organisational interventions for caregivers, as well as the need to develop solutions that enable a more equitable distribution of caregiving responsibilities in the family.

Age was not found to have a significant effect on the degree of emotional, instrumental, or informational support (Table 5).

The analysis of the relationship between caregivers’ age and the support that they provide indicated that higher levels of buffering (protective) support were more common among caregivers aged over 45 years. This pattern may be indicative of differences in caregiving at various stages of life. Younger caregivers are more likely to combine caregiving with paid employment and other responsibilities, which can limit their capacity to take on additional caregiving tasks. On the other hand, older caregivers—benefiting from greater life experience and more stable occupational or family circumstances—may be better positioned to organise and safeguard care.

Age-related differences in support provision point to the need to tailor forms of assistance to the specific circumstances of different age groups. Younger caregivers may need support that helps them balance caregiving with work, whereas support for older caregivers may be better directed towards maintaining physical capacity and accessing respite services. Overall, these findings reveal that caregivers’ age is an important factor shaping both the type of support provided and the strategies employed in daily care.

The burden associated with informal caregiving was substantial. The most frequently reported difficulties were psychological fatigue (70.9%) and physical fatigue (60.2%). Caregivers also indicated limited access to reimbursed healthcare services and the lack of temporary replacement in caregiving as major external barriers, highlighting the cumulative and persistent nature of caregiving burden.

Overall, while chi-square analyses suggested selected gender-related differences in emotional, instrumental, and protective support, these findings should be interpreted cautiously, as sensitivity analyses using Fisher’s exact test attenuated statistical significance in some comparisons.

## 4. Discussion

Caring for people with neurological conditions is highly demanding, both physically and emotionally. These conditions—chronic, progressive, and unpredictable—disrupt fundamental aspects of human functioning and lead to a gradual loss of independence. As a result, caregivers take on many roles. In addition to providing day-to-day care, they often become the main organisers of the care recipient’s daily life, as well as their advocates, companions, and sources of emotional support [3,18]. Research increasingly recognises caregiving as a complex and deeply human experience, involving care, constant vigilance, and a form of responsibility that goes beyond routine tasks. This complexity is well described in established theoretical models, which help explain the psychosocial consequences of the caregiving role [19,20,21].

In the Polish context, long-term care remains largely dependent on informal caregivers, who provide the majority of daily support for individuals with chronic neurological conditions. Although selected financial benefits and caregiver allowances are available, access to coordinated institutional support remains limited, particularly with regard to respite care services and temporary replacement in caregiving. Moreover, healthcare and social care systems operate in a fragmented manner, which places an additional organisational burden on families. As a result, informal caregivers frequently assume extensive responsibilities with insufficient professional support, increasing the risk of cumulative physical and psychological strain. These systemic characteristics are essential for interpreting the high levels of burden observed in the present study.

Pearlin’s stress process model suggests that caregiver burden arises from the interaction between primary stressors—such as disease symptoms, loss of independence, and behavioural disturbances—and secondary stressors, including role conflicts, financial difficulties, institutional barriers, and emotional strain. At the same time, caregivers may draw on resources that help mitigate stress, including social support, a sense of meaning, and caregiving skills. Similarly, Lazarus and Folkman’s transactional model of stress and coping posits that individuals’ subjective appraisal of a situation—whether it is seen as a threat, a loss, or a challenge—determines the choice of coping strategies and the perceived level of burden. From this perspective, the support that caregivers provide serves two functions: it meets the needs of the person receiving care and helps caregivers regulate their own emotions and maintain psychological balance in a highly demanding situation.

The findings of this study clearly show that caregivers of people with neurological conditions provide support of high intensity. The vast majority of respondents reported moderate to high levels of emotional, instrumental, and informational support. The particularly high level of emotional support highlights the central importance of relationships in caregiving: presence, patience, understanding, and companionship form the basis of the care recipient’s sense of safety and security [22].

Instrumental support—such as assistance with personal care, physical transfers, medication administration, and organising daily routines—places a substantial physical demand on caregivers. This observation is consistent with previous research on caregivers of people after stroke, with Parkinson’s disease, and with dementia [16]. Informational support, although provided to a lesser extent than emotional and instrumental support, illustrates caregivers’ active efforts to seek knowledge needed for everyday decision-making related to treatment and care organisation.

High levels of support—particularly emotional and instrumental—were more commonly reported by women. This pattern has been widely documented in the literature, which shows that women more often assume caregiving roles, tend to be more emotionally involved, and have greater experience with caregiving tasks. At the same time, research also indicates that women are more vulnerable to prolonged emotional fatigue, feelings of overload, and caregiver burnout. This supports the case for targeted support measures for female caregivers. Age-related differences were also observed. Older caregivers reported higher levels of buffering (protective) support, which may be associated with higher emotional stability, broader life experience, and more established ways of coping. These findings are consistent with both Pearlin’s stress process model and the transactional model of stress and coping proposed by Lazarus and Folkman [19,23]. However, sensitivity analyses using Fisher’s exact test attenuated the statistical significance of these associations, highlighting the exploratory nature of the observed gender-related patterns.

Psychological fatigue emerges from the analysis as the most significant burden for caregivers. It is linked to chronic stress, a constant sense of responsibility, the unpredictable progression of neurological illness, and limited opportunities for rest. Physical fatigue, in turn, results from the frequent performance of repetitive and physically strenuous caregiving tasks that require strength and endurance. These findings are consistent with international research showing that caregivers of people with neurological disorders experience greater burden than caregivers in many other disease groups [24].

Importantly, the reported burden cannot be interpreted as incidental or short-term. The high prevalence of both psychological and physical fatigue, combined with structural barriers such as limited access to reimbursed healthcare services and the lack of respite care, suggests that caregiver burden in this population is persistent and cumulative in nature. This pattern is consistent with previous research indicating that long-term neurological caregiving often leads to chronic overload rather than transient stress.

External factors that make caregiving more difficult are also of great importance. The main barrier reported by respondents was limited access to publicly funded healthcare services. This included long waiting times, a shortage of available rehabilitation appointments, and difficulties in accessing specialist care. European research identifies inadequate institutional support and limited access to respite services as persistent problems, particularly in Central and Eastern European countries. Another major barrier was the absence of someone who could temporarily take over caregiving duties, which increases caregivers’ feelings of isolation and constant strain. Gaps in information—such as difficulties understanding benefit systems and the absence of clear and accessible support pathways—further add to uncertainty and confusion.

Caregivers’ preferences regarding forms of respite support clearly reflect their everyday needs. The most frequently indicated option was day care for the care recipient at a support centre, which points to a strong need for relief, rest, and recovery time. Access to information and education was also seen as highly important. These areas are often overlooked in healthcare systems, despite their crucial role in strengthening caregivers’ sense of competence and ability to cope with their responsibilities. Support groups, psychological counselling, and services provided by a Disability Support Assistant were mentioned less often, but still attracted notable interest [20,24,25,26,27].

These findings show that caregivers continue to operate within a system that fails to provide sufficient formal or informational support. Their strong commitment, despite institutional shortcomings, demonstrates the considerable social value of informal caregivers. At the same time, the results underline the need for strategies that strengthen caregivers’ resources and reduce the negative effects of caregiving burden. This is essential not only for caregivers’ wellbeing, but also because the quality of informal care is one of the most important factors influencing the quality of life of people living with neurological conditions [28,29,30].

### 4.1. Implications for Practice

1. Expansion of respite care services, including both day care and 24 h options.
  Caregivers clearly need opportunities for temporary relief from caregiving. Access to respite care can substantially reduce the risk of physical and emotional exhaustion.
2. Improved access to rehabilitation, neurological consultations, and publicly funded healthcare services.
  Limited availability of these services was one of the most frequently reported barriers. Improving access may reduce waiting times, ease the burden on caregivers, and increase the effectiveness of formal care.
3. Strengthening information and educational support systems.
  Caregivers require clear, reliable, and practical information about the illness, available benefits, medical equipment, and care organisation. Closing information gaps may reduce uncertainty and enhance caregivers’ confidence and sense of competence.
4. Provision of psychological support and support groups.
  Although not the most commonly selected forms of assistance, psychological support and peer groups can make a difference in preventing caregiver burnout and supporting emotional wellbeing.
5. Training and educational programmes for caregivers, focusing on home-based care, managing crisis situations, navigating the healthcare system, and using medical and rehabilitation equipment.
6. Development of coordinated, interdisciplinary care pathways that consider the needs of both care recipients and caregivers, in line with Integrated Care initiatives promoted across Europe.

### 4.2. Strengths and Limitations of This Study

A major strength of this study is its focus on the support that caregivers actually provide, rather than only on their perceived needs or subjective assessments. This approach is used less often in research, but it provides a clearer picture of what caregiving involves in everyday practice. Another important strength is the use of proxy reporting, which combines information provided by caregivers with data on the health and functioning of the care recipients. This makes it possible to interpret the findings in relation to the clinical characteristics of the person receiving care, which is rarely carried out in studies involving people with different neurological conditions.

This study also gains value from being conducted in the context of the Polish healthcare system, where research on caregivers of people with neurological conditions is still limited. The findings help fill an important gap in knowledge and may inform future research, service planning, and policy development.

The main limitation of this study is its cross-sectional design, which restricts the ability to assess how caregiving changes over time or how it relates to disease progression. In addition, the data are based on self-report, which carries a risk of bias stemming from caregivers’ perceptions. Although this limitation is common in survey-based studies, it should be considered when interpreting the results. Additionally, sparse data in some categories and the relatively small number of male caregivers limited statistical power; therefore, selected group comparisons should be interpreted with caution.

Several limitations of this study should be acknowledged. First, the use of proxy reporting may have introduced reporting bias, as caregivers’ assessments could have resulted in either overestimation or underestimation of the level of support provided. Nevertheless, proxy reporting is commonly used in studies involving neurological populations and was considered appropriate given the frequent presence of cognitive and communication impairments among care recipients.

Second, although a minimum caregiving duration of one month was applied as an inclusion criterion, detailed quantitative data on the total duration of caregiving were not collected in a form that would allow for stratified analyses. Consequently, the relationship between caregiving duration and the level of burden could not be examined. Future studies should include caregiving duration as a key variable to better capture temporal patterns of caregiver burden.

## 5. Conclusions

Caregivers of people with neurological conditions provide ongoing, intensive support, especially in emotional and practical domains, underscoring their central role in enabling the daily functioning and safety of care recipients.

Informational support is provided less often, largely due to poor access to reliable information and the complexity of the healthcare system. This finding demonstrates a clear and pressing need for better education and guidance for caregivers.

Women more frequently reported providing high-intensity care than men, reflecting well-documented gender patterns in caregiving roles; however, these differences should be interpreted with caution. This finding suggests that gender should be considered when planning support interventions for caregivers. Older caregivers tended to report higher levels of buffering (protective) support, which may be associated with greater life experience and more developed coping strategies. Psychological and physical exhaustion are the most serious difficulties reported by caregivers, resulting from long-term burden and insufficient opportunities for rest. These findings clearly support the need to expand and prioritise respite care services.

Limited access to healthcare services and the absence of temporary replacement care were identified as major barriers to effective caregiving. Addressing these challenges requires improved availability of social services and better coordination between health and social care systems.

Overall, caregivers’ preferences regarding respite support—such as day care for care recipients and improved access to information about available benefits—indicate priority areas for system-level actions with the potential to reduce caregiver burden and enhance the sustainability of informal care.

## Figures and Tables

**Table 1:** Sociodemographic characteristics of the caregiver sample.

Variable	Category	n	%
Gender	female	89	86.4%
	male	14	13.6%
Age (years)	18–35	28	27.2%
	36–45	17	16.5%
	46–55	39	37.9%
	≥56	19	18.4%
Marital status	single	24	23.3%
	married	73	70.9%
	informal partnership	1	1.0%
	widowed	3	2.9%
	divorced	2	1.9%
Place of residence	rural	68	66.0%
	urban	35	34.0%
Education	primary	0	0.0%
	vocational	8	7.8%
	secondary	39	37.9%
	higher (tertiary)	56	54.4%
Main source of income	paid employment	89	86.4%
	old-age pension	6	5.8%
	disability benefit	1	1.0%
	financially dependent on family	7	6.8%

**Table 2:** Socioclinical characteristics of the patient (care recipient) sample.

Variable		n	%
Age (years)	≤60	24	23.3%
	61–70	23	22.3%
	71–80	28	27.2%
	≥81	28	27.2%
Functional status	Fully dependent on others	39	37.9%
	Partially dependent on others	36	35.0%
	Requires minimal assistance with self-care	28	27.2%
Primary diagnosis	Stroke (post-stroke condition)	39	37.9%
	Epilepsy	5	4.9%
	Dementia	34	33.0%
	Parkinson’s disease	8	7.8%
	Meningitis	1	1.0%
	Multiple sclerosis	6	5.8%
	Amyotrophic lateral sclerosis	1	1.0%
	Spina bifida	1	1.0%
	Spinal muscular atrophy	1	1.0%
	Huntington’s disease	1	1.0%
	other	6	5.8%

**Table 3:** Social support provided by caregivers (BSSS subscales).

Statistic	Currently Provided Emotional Social Support (0–27 Points)	Currently Provided Instrumental Social Support (0–9 Points)	Currently Provided Informational Social Support (0–6 Points)	Satisfaction with Currently Received Social Support (0–3 Points)	Buffering (Protective) Support (0–18)
Mean	21.44	7.26	4.19	1.99	12.42
Median	22.00	8.00	4.00	2.00	13.00
SD	4.68	1.79	1.58	0.88	3.87
Minimum	10.00	0.00	0.00	0.00	0.00
Maximum	27.00	9.00	6.00	3.00	18.00

**Table 4:** Social support provided by caregivers by gender.

Variable		Gender				*p*
		Women		Men		
		n	%	n	%	
Currently provided emotional social support	low	0	0.0%	0	0.0%	χ^2^ = 7.738; *p* = 0.0054
	moderate	24	72.7%	9	27.3%	
	high	65	92.9%	5	7.1%	
Currently provided instrumental social support	low	3	75.0%	1	25.0%	χ^2^ = 7.175; *p* = 0.0277
	moderate	22	73.3%	8	26.7%	
	high	64	92.8%	5	7.2%	
Currently provided informational social support	low	14	77.8%	4	22.2%	χ^2^ = 1.452; *p* = 0.484
	moderate	34	87.2%	5	12.8%	
	high	41	89.1%	5	10.9%	
Satisfaction with currently received social support	low	21	80.8%	5	19.2%	χ^2^ = 0.972; *p* = 0.615
	moderate	40	88.9%	5	11.1%	
	high	28	87.5%	4	12.5%	
Buffering (protective) support	low	3	75.0%	1	25.0%	χ^2^ = 0.981; *p* = 0.6122
	moderate	37	84.1%	7	15.9%	
	high	49	89.1%	6	10.9%	

**Table 5:** Social support provided by caregivers by age.

Variable		Age				*p*
		≤45 Years		>45 Years		
		n	%	n	%	
Currently provided emotional social support	low	0	0.0%	0	0.0%	χ^2^ = 2.117; *p* = 0.1457
	moderate	11	33.3%	22	66.7%	
	high	34	48.6%	36	51.4%	
Currently provided instrumental social support	low	2	50.0%	2	50.0%	χ^2^ = 0.274; *p* = 0.8721
	moderate	12	40.0%	18	60.0%	
	high	31	44.9%	38	55.1%	
Currently provided informational social support	low	7	38.9%	11	61.1%	χ^2^ = 0.266; *p* = 0.8756
	moderate	18	46.2%	21	53.8%	
	high	20	43.5%	26	56.5%	
Satisfaction with currently received social support	low	9	34.6%	17	65.4%	χ^2^ = 1.399; *p* = 0.4969
	moderate	20	44.4%	25	55.6%	
	high	16	50.0%	16	50.0%	
Buffering (protective) support	low	3	75.0%	1	25.0%	χ^2^ = 6.388; *p* = 0.041
	moderate	24	54.5%	20	45.5%	
	high	18	32.7%	37	67.3%	

## Data Availability

Data can be obtained by contacting the corresponding author.

## Abbreviations

The following abbreviations are used in this manuscript:

BSSS	Berlin Social Support Scales
